# Potential mechanisms of quercetin in cancer prevention: focus on cellular and molecular targets

**DOI:** 10.1186/s12935-022-02677-w

**Published:** 2022-08-15

**Authors:** Parina Asgharian, Abbas Pirpour Tazekand, Kamran Hosseini, Haleh Forouhandeh, Tohid Ghasemnejad, Maryam Ranjbar, Muzaffar Hasan, Manoj Kumar, Sohrab Minaei Beirami, Vahideh Tarhriz, Saiedeh Razi Soofiyani, Latipa Kozhamzharova, Javad Sharifi-Rad, Daniela Calina, William C. Cho

**Affiliations:** 1 Drug Applied Research Center, Tabriz University of Medical Sciences, Tabriz, Iran; 2 Department of Pharmacognosy, Faculty of Pharmacy, Tabriz University of Medical Sciences, Tabriz, Iran; 3 Department of Biochemistry and Clinical Laboratories, Faculty of Medicine, Tabriz University of Medical Sciences, Tabriz, Iran; 4 Student Research Committee, Shiraz University of Medical Sciences, Shiraz, Iran; 5 Department of Molecular Medicine, Faculty of Advanced Medical Sciences and Technologies, Shiraz University of Medical Sciences, Shiraz, Iran; 6 Molecular Medicine Research Center, Biomedicine Institute, Tabriz University of Medical Sciences, Tabriz, Iran; 7 Agro Produce Processing Division, ICAR—Central Institute of Agricultural Engineering, Bhopal, 462038 India; 8 Chemical and Biochemical Processing Division, ICAR—Central Institute for Research on Cotton Technology, Mumbai, 400019 India; 9 Department of Biochemistry, School of Medicine, Iran University of Medical Sciences, Tehran, Iran; 10 Clinical Research Development Unit of Sina Educational, Research, and Treatment Center, Tabriz University of Medical Sciences, Tabriz, Iran; 11 Scientific Production and Technical Center Zhalyn, А20Х3F6 Almaty, Kazakhstan; 12 Facultad de Medicina, Universidad del Azuay, 14-008 Cuenca, Ecuador; 13 Department of Clinical Pharmacy, University of Medicine and Pharmacy of Craiova, 200349 Craiova, Romania; 14 Department of Clinical Oncology, Queen Elizabeth Hospital, Kowloon, Hong Kong, China

**Keywords:** Quercetin, Malignant tumors, Pharmacology, Signalling pathways, Molecular targets

## Abstract

Over the past few years, the cancer-related disease has had a high mortality rate and incidence worldwide, despite clinical advances in cancer treatment. The drugs used for cancer therapy, have high side effects in addition to the high cost. Subsequently, to reduce these side effects, many studies have suggested the use of natural bioactive compounds. Among these, which have recently attracted the attention of many researchers, quercetin has such properties. Quercetin, a plant flavonoid found in fresh fruits, vegetables and citrus fruits, has anti-cancer properties by inhibiting tumor proliferation, invasion, and tumor metastasis. Several studies have demonstrated the anti-cancer mechanism of quercetin, and these mechanisms are controlled through several signalling pathways within the cancer cell. Pathways involved in this process include apoptotic, p53, NF-κB, MAPK, JAK/STAT, PI3K/AKT, and Wnt/β-catenin pathways. In addition to regulating these pathways, quercetin controls the activity of oncogenic and tumor suppressor ncRNAs. Therefore, in this comprehensive review, we summarized the regulation of these signalling pathways by quercetin. The modulatory role of quercetin in the expression of various miRNAs has also been discussed. Understanding the basic anti-cancer mechanisms of these herbal compounds can help prevent and manage many types of cancer.

## Introduction

Cancer is a set of diseases in which cells develop abnormally and have the ability to invade and spread (metastasize) to other parts of the body [1, 2]. Chemotherapy drugs on the market today are effective in treating cancer, but they are expensive and come with a long list of undesirable side effects [3, 4]. Chemopreventive properties of natural bioactive substances have been increasingly important to researchers in the field of medicine in recent years [5, 6]. More and more research has demonstrated the anti-cancer potential and usefulness of natural ingredients in the treatment of cancer [7–10]. Quercetin, a plant flavonol produced from the polyphenol family, is an example of a useful, accessible, and highly effective natural substance. It is found at great levels in fruits, vegetables, leaves, and seeds [11]. Quercetin has many physiological activities such as antioxidant and free radical scavenger [12], iNOS synthase inhibitor [13], xanthine oxidase inhibitor [14, 15], reduction of leukocyte immobilization [16], and modulation of gene expression [17, 18]. Numerous studies have shown that quercetin is used to treat a variety of diseases including coronary heart diseases [19], diabetes [20] and cancers [21, 22].

The anti-cancer effects of quercetin have been investigated in many studies and the role of that in preventing the growth, proliferation and progression of cancer through cellular signalling pathways such as Wnt/β-catenin signalling, phosphoinositide 3-kinase (PI3K)/protein kinase B (AKT) pathway, Janus kinase (JAK)/signal transducer and transcription activator (STAT) signalling pathway, mitogen-activated protein kinase (MAPK) pathway, p53 signalling and nuclear factor kappa B (NF-κB) pathway [23]. It was also demonstrated that quercetin can impact and specifically target tumour suppressor and oncogenic miRNAs and lncRNAs [24–26].

An important therapeutic limitation is due to the low absorption of quercetin; therefore, researchers have been looking for solutions to increase plasma concentrations such as incorporation into nanoparticles or structural chemical changes [27]. The anticancer effects of nanoparticles have been confirmed in a recent study conducted by Lou et al., The results showed that quercetin nanoparticles induced cell death in human neuroglioma cells in a dose-and time-dependent manner; it also markedly promoted apoptosis in the cells [28]. Besides, the results revealed the positive correlation of ERK, LC3, cleaved Caspase-3, cytoplasm p53, and PARP expressions with quercetin nanoparticle concentrations [28]. The results also showed the induction of apoptosis and autophagy by quercetin nanoparticles is at least partly through the activation of LC3/ERK/Caspase-3 and the inhibition of AKT/mTOR signalling pathways [28]. Another possibility of increasing the bioavailability of quercetin is encapsulation in phytosomes. Phytosomes are derived from phospholipids, which are made from the same cell membrane material. When encapsulated in a phytosome, quercetin can cross the cell membrane and is delivered directly inside the cell. By combining this compound with a unique phospholipid delivery system, the quercetin in this formula is more bioavailable. The technology used is similar to liposomal encapsulation technology [29].

Isoquercitrin is an enzymatically modified form of Quercetin with increased bioavailability, a bioflavonoid that has significant antiallergic effects and contributes to immune function. Enzymatically modified isoquercitrin is prepared using a natural enzymatic process that attaches polysaccharides to convert quercetin, which has poor bioavailability, into a water-soluble form (Alpha-Glycosyl Isoquercitrin). These are the top benefits of quercetin, such as high absorption and increased bioavailability. According to pharmacokinetic data, the absorption of isoquercetin is up to 40 times higher (C_max_) than that of quercetin and reaches maximum levels in the bloodstream in just 15 min [30]. Isoquercitrin has been used in a few clinical studies studying the treatment of kidney cancer, renal cell carcinoma, advanced renal cell carcinoma, and venous thromboembolism in pancreatic cancer and colorectal cancer. It has antineoplastic activity and has been shown to reduce the rate of polymerization of red blood cells. It acts as an antineoplastic agent, bone density preservative, osteogenesis regulator, antioxidant, histamine antagonist and antipruritic drug. As a result, isoquercitrin is much more active than quercetin and has a wider range of therapeutic activities [31]. Another limitation of this update is represented by a lack of clinical studies that support its anticancer effect. A clinical study on 144 adenocarcinoma (AD) and 120 squamous cell carcinoma (SQ) patients has revealed that a quercetin-rich diet can highly influence the expression of the miRs. Let-7 family and miR-146a which act as tumor suppressors have significantly higher expression in more quercetin consumer lung cancer patients. On the other side, miR-17 oncogenic shows downregulation in 67% of its members [32].

The purpose of this review is to examine quercetin's function in cancer through influencing cellular signalling pathways, miRNA and lncRNA expression. With a thorough understanding of the mechanism behind quercetin's anti-cancer actions, it is possible to conclude that this combination might be employed clinically in cancer therapy.

## Review methodology

To conduct this comprehensive review, we searched numerous specialized electronic databases for research on the anticancer and chemopreventive properties of quercetin, including PubMed/Medline, DynaMed Plus, Embase, ScienceDirect, and the TRIP database. The following MeSH terms were used in the literature search: "antineoplastic agents," "phytogenic/pharmacology," "antioxidants/pharmacology," "cell line," "tumour," "diet," "humans," "neoplasms/drug treatment," and "quercetin/pharmacology." There were no constraints on language or publication type, and the reference lists of chosen papers were carefully examined, omitting research including homoeopathic medications.

Inclusion criteria include the following: (i) preclinical studies on quercetin's anticancer effects; (ii) in vitro studies on cancer cells with evidence of molecular processes, and (iii) in vivo animal studies using well-defined experimental dosages.

Exclusion criteria include the following: (i) abstracts, incomplete articles, and duplicate articles; (ii) studies that did not establish a direct correlation between the anticancer effect and the observed results; and (iii) studies that did not use quercetin alone as an intervention group and instead used it in combination with other cytostatic drugs.

## Anti-cancer effects of quercetin: modulation of cancer signalling pathways

### Wnt/β-catenin pathway

Various physiological processes such as cell proliferation, differentiation, stemness, migration, and apoptosis are controlled by the Wnt signalling system, also known as APC/β-catenin/Tcf pathway [33].

There are two different Wnt/β-catenin signalling pathways: canonical and non-canonical. Canonical pathway employ β-catenin regulating gene expression, in contrast, non-canonical is a β-catenin-independent pathway [34]. To be more specific, in the canonical pathway, Wnt ligand (Wnt1 and Wnt3a) binds to Frizzled and Lrp5/6 receptor complexes which lead to stabilization and transcriptional activation of cytoplasmic β-catenin while non-canonical Wnt signalling pathway is represented as a response to Wnt ligands which is independent of β-catenin stabilization [35]. The loss of Wnt signalling regulation is frequently correlated with tumor progression and metastasis [36]. Various studies have demonstrated that the anti-tumor effect of quercetin on the Wnt/β-catenin pathway has a multifunctional impact. Cells from the NT2/D1 line were studied by Mojsin et al. (human in vitro model of teratocarcinoma). In NT2/D1 cells, they discovered that quercetin inhibits SOX2, Nanog, and Oct4 expression, as well as β-catenin nuclear translocation, resulting in a decrease in β-catenin-dependent transcriptional activity [37]. In research by Haesung et al., quercetin caused apoptosis in 4T1 cells and showed dose-dependent anticancer efficacy (murine mammary cancer cell line). After treatment with 20 µM quercetin, Dickkopf-related protein 1 (DKK1), 2 and 3, which are regulators of Wnt signalling, are increased and cell viability is reduced, according to the researchers [38]. The Wnt signalling pathway was studied in human colon cancer cells (SW480) by Shan et al., who found that quercetin inhibits the production of cyclin D1 and survivin, which are involved in the cell cycle regulation and death, in the cells [39]. Quercetin's influence on GSK3 has been examined in a recent study on HT29 colon cancer cells (key elements of the Wnt pathway). Quercetin at doses of 10 to 75 µM did not significantly inhibit GSK3 and GSK3, and as a result, the total β-catenin level in HT29 cells was mostly unaffected. Various experimental setups and biological settings can produce different kinds of reactions [40].

### The PI3K/AKT pathway

For AKT to translocation to the plasma membrane, phosphatidylinositol-3-kinase (PI3K) has to be present in the cell. Biochemical processes such as cell cycle progression, differentiation, cell survival, and cell proliferation are all influenced by the PI3K/AKT pathway [41]. The Bcl-2 protein family and Bax are controlled by the PI3K/AKT pathway, which has anti-apoptotic properties (a pro-apoptotic gene) [42]. Deregulation of this pathway could be a key event in cancer pathogenesis [41]. Several studies explored the quercetin effect on PI3K/AKT pathway. Using the HCC1937 PTEN-null cancer cell line, Gulati et al. observed that 25 µM quercetin may decrease active AKT/PKB phosphorylation and dramatically limit cell proliferation of PTEN-null cancer cells, which is consistent with previous studies [43].

In a study on HL-60 leukaemia cell lines, Yuan et al. investigated the quercetin mechanism and discovered that quercetin decreased p-AKT and Bcl-2 levels, as well as triggered apoptosis, in a manner that was associated with the inhibitory actions of PI3K/AKT. It was revealed that at 150 µM quercetin, the inhibitory effects were the most pronounced [42, 44, 45].

### JAK/STAT signalling pathway

In interconnected networks in a cell, the JAK/STAT (Janus kinase/signal transducers and activators of transcription) signalling pathway detects stimulus signals from outside the cell and transmits its message to the cell nucleus and activates several transcription factors [46–48]. The JAK/STAT pathway is involved in regulating processes such as immune maintenance, cell division and growth, cell death, and tumor formation. JAK/STAT pathway components are regulated by other paths such as ERK MAPK and PI3K [49–51]. Skin, immune system, and cancer disorders are caused by JAK/STAT pathway disruptions [52].

In line with this, Qin et al. examined the effect of quercetin on leptin and its receptor in MGC-803 cells via the JAK/STAT signalling pathway. They found that quercetin caused the arrest of cells in the G2/M stage of the cell cycle through the p-STAT3 pathway and celled to apoptosis and necrosis. On the other hand, the flavonoid compound reduces the expression of leptin and its receptor [53].

It has been shown that quercetin can inhibit IL-6-induced glioblastoma cell growth and migration by regulating the STAT3 signalling pathway, which affects the expression of this protein in glioblastoma cells. In T98G and U-87 cell lines, quercetin blocked the IL-6-induced STAT3 pathway, reducing the expression of GP130, JAK1, and STAT3. As a result, there is a reduction in the proliferation and migration of cancer cells [54].

The effects of quercetin and epigalactochin-3-gallate (EGCG) on cholangiocarcinoma cells were investigated by Senggunprai et al. IL-6 and IFN-gamma were found to regulate JAK/STAT (STAT1/3 phosphorylation) pathways in cholangiocarcinoma cells, and the results suggested that these two chemicals can be employed as chemopreventive agents against these cells. Lastly, these compounds were able to suppress KKU100 cancer cell proliferation and migration [55, 56].

Quercetin suppresses clonogenic survival in BT-474 cells, triggers apoptosis via caspase 3, 8, and PARP cleavage, and causes cell cycle arrest in the sub-G0/G1 phase, according to a study by Seo et al., which also found that quercetin reduces p-JAK1 and p-STAT3 expression and inhibits MMP-9 secretion. When it comes to HER-2-expressing breast cancer, this flavonoid can both prevent and treat the disease [57]. Luo et al. examined the processes of apoptosis, autophagy, and quercetin proliferation in cervical cancer cells. Using quercetin-conjugated gold nanoparticles, they found that the complex shows a similar role in suppressing JAK2, a protein expressed in cervical cancer cells, which suppresses proliferation, invasion, and migration processes. They also found that apoptosis and autophagy processes occurred through caspase-3 in cancer cells inhibited by JAK2, and that cyclin D1 and mTOR were suppressed by the STAT3/5 and PI3K/AKT signalling pathways [58, 59].

### The MAPK signalling pathway

Mitogen-Activated Protein Kinase (MAPK) fall into three main categories: ERKs (stimulated by mitogens and growth factors), JNK/SAPK, and p38s (stimulated by cellular stress and inflammatory cytokines) [60–62]. Cell proliferation, differentiation, gene expression, cell survival, mitosis, and apoptosis are all affected by MAPK, which has been found in MAPK14, MAPK 7, and MAPK 12 [63]. Due to the association between the MAPK pathway and many types of cancer, some bioactive natural compounds, such as quercetin, have been found to prevent cancer by altering the MAPK signalling pathway. Chen et al. observed that stimulating the p38 MAPK signalling pathway boosted the expression of ABCG2, MDR1, and pHSP27 genes in multidrug-resistant spheres of oral cancer cells. Through the suppression of pHSP27 expression and the progressive modification of EMT, they discovered that quercetin might promote apoptosis in cancer spheres. Quercetin and cisplatin, when combined, can inhibit the development of oral cancers and diminish the resistance of the malignancies to treatment [64].

The isocourestine chemical isolated from Bidens bipinnata L. was examined by Huang et al. and was proven to inhibit liver tumour growth and progression. Activation of caspases 3, 8, and 9 leads to an increase in apoptosis, which in turn triggers JNK phosphorylation, suppresses the production of ERK and p38 MAPK and lowers the level of PKC, all of which lead to the cell cycle arrest at G1. Isoquercetin, on the other hand, inhibits the development of transplanted tumours in naked mice when tested in vivo [65–67]. Zhu et al., studied the molecular effect of a quercetin derivative called 7-O-granuestine quercetin (GQ) on gastric cancer cells (SGC-7901 and MGC-803). They found that the compound induced cancer cell apoptosis and cell arrest in the G2/M phase. Its molecular mechanism is that GQ increases ROS production, activates the p38 and JNK pathways, disrupts the regulation of Bcl-2, Bcl-xl, and Bax proteins, releases cytochrome c, and ultimately induces apoptosis [68, 69]. ERK and AKT were shown to be connected with the advancement of esophageal squamous cell carcinoma in a study conducted by Zhao et al. The phosphorylated form of these proteins was found to be associated with the progression of the disease. Quercetin-3-methyl ether was employed to limit the kinase activity of these proteins, which in turn inhibited the proliferation and advancement of esophageal cancer cells, according to the researchers [70].

Another study on prostate cancer (PC3 and LNCaP cell line) by Erdogan et al., showed that quercetin inhibited the proliferation of PC3 and CD44^+^ / CD133^+^ cells. On the other hand, co-administration of siRNA and quercetin induces apoptosis and induces cell entry into G1 and cell arrest, and finally inhibits the phosphorylation of AKT, PI3K, and ERK1/2 proteins and the expression of p38, ABCG2 proteins, and NF-κB was reduced [71–73].

The apoptotic effects of quercetin on canine osteosarcoma cells (D17 and DSN) were examined by Ryu et al., Apoptosis, cell cycle, ROS content, mitochondrial membrane depolarization, intracellular calcium concentrations, and quercetin's antiproliferative effect were all shown to be affected by quercetin. S6, AKT, and p70S6K proteins can be inhibited by quercetin whereas ERK1/2, p38, and c-JNK proteins can be elevated. Osteosarcoma cells die as a result of the combination of these events [74].

### The p53, NF-ĸB, and apoptotic pathways

In human CRC cell lines obtained from patients with microsatellite instability (MSI), quercetin stimulated 5-fluorouracil-induced apoptosis in a p53-dependent way [75]. Bcl-2 protein expression was reduced in CO115 cells when quercetin and 5-FU were combined. This suggests that quercetin and 5-FU synergism is dependent on the apoptotic mitochondrial pathway [75]. HCT15 (p53 mutant) cells, on the other hand, lacked this synergy, indicating that quercetin's effects are p53-dependent [75]. Quercetin also accelerated the apoptosis generated by doxorubicin (DOX) in hepatoma cells in a p53-dependent manner by downregulating Bcl-xl expression [76]. In line with this result, the quercetin impact on DOX-mediated apoptosis was reduced upon the applying pifithrin-a (p53 inhibitor), Z-VAD fmk (caspase inhibitor), or Bcl-xl expression vector [76]. Moreover, when Bcl-xl expression is inhibited, quercetin promoted p53 protein expression, Bax translocation, transcriptional activity and DOX-induced PUMA (p53 upregulated modulator of apoptosis) expression [76]. Quercetin promoted the expressions of cell death-related genes including Bax, p53, caspase-3 and cytochrome-c in cervical cancer cells, while significantly down-regulated AKT and Bcl-2 expression [77]. Quercetin promoted mitochondrial cytochrome-c release and the accumulation of ROS, resulting in apoptosis and cell cycle arrest at G2/M phases [77]. It has been shown that quercetin strongly promoted p53, Sestin 2, and activated AMPK protein expressions while suppressing mTOR in a dose-dependent manner [78]. Quercetin generated intracellular ROS, leading to the induction of apoptosis and the regulation of the Sestrin 2/AMPK/mTOR pathway in a p53- independent manner [78]. It has been reported that quercetin significantly enhanced Trichostatin A (TSA)-induced apoptosis and growth arrest in A549 cells; the transfection of p53 siRNA reduced its enhancing impacts [79]. However, since p53 silencing does not entirely limit quercetin's effect on TSA-induced apoptosis in A549 cells, it has been postulated that the p53-independent route may potentially contribute to quercetin's boosting effect [79]. Quercetin promoted the antitumor effect of TSA in a xenograft mouse model of lung cancer [79]. The combination of TSA and quercetin treatment of tumors in mice increased p53 and apoptosis levels [79]. It has been reported that quercetin, regardless of p53 status, markedly suppresses cell proliferation, and promotes sub-G1 and apoptotic cell populations in lung cancer cells [80]. Besides, upon treatment of H460 cells with quercetin, genes related to death pathways such as the c-Jun Nterminal kinase (JNK) pathway (JNK, MEKK1, MKK4), death receptors (TNFR1, TRAILR, FAS), the interleukin-1 receptor pathway (IRAK, IL1, IL1R), the NF-κB pathway (IκBα) and the caspase cascade (dFF45, caspase-10) were upregulated, while genes associated with cell survival (NF-κB, AKT, IKK) and genes involved in cell proliferation (SCF, CdKs, SKP2, cyclins) were downregulated [80, 81]. Moreover, quercetin promoted the expression of GAdd45, involved in cell cycle arrest [80].

Quercetin generates intracellular ROS production, decreases mitochondrial membrane potential, and promotes sestrin 2 expression via the AMPK/p38 pathway, thus leading to apoptosis in HT-29 colon cancer cells in a p53-independent pathway [82]. Nano-quercetin (NQ) has been shown to activate p53-ROS crosstalk and cause apoptosis in HepG2 cells; in addition, it triggers epigenetic modifications that lead to cell cycle arrest in the sub-G phase, decreased cell populations in the mitotic and synthetic phases, and suppression of proliferation [83]. In addition, MET containing a variety of phenolics such as quercetin, homoorientin, naringin, and isorhamnetin has been found to induce apoptosis in HCCSCs without relying on p53 by increasing the Bax/Bcl-2 ratio; cleaved PARP protein expression was used to indicate apoptosis [84, 85].

Quercetin caused a marked cell cycle arrest in HT-29 cells in the S-phase [86]. A reduction in phosphorylated-AKT and CSN6 protein expression, as well as a decrease in Bcl-2 and Myc protein expression, was seen after quercetin administration [86]. Moreover, CSN6 overexpression omitted the impacts of quercetin treatment on HT-29 cells, indicating the involvement of the AKT-CSN6-Myc signalling axis in quercetin-induced apoptosis in the cells [86]. The synergistic effect of rutin (a glycoside with 5-FU( in the induction of apoptosis in PC3 cells line has been reported [87]. The combination of rutin/5-FU promoted p53 gene expression and decreased Bcl-2 protein expression [87]. A quercetin-supplemented diet promoted the antitumor effect of TSA in nude mice which bears lung cancer in a dose-dependent manner via the upregulation of p53 [88].

Cell cycle arrest and apoptosis have been seen in MDA-MB-231 breast cancer cells after treatment with quercetin, which was decreased by knocking down the protein Foxo3a [89]. Furthermore, quercetin-induced Foxo3a activity was eliminated by using a JNK inhibitor [89]. The activities of p21, p53 and GADD45 signalling pathways, activated by quercetin, were decreased as a result of Foxo3a knockdown and the suppression of JNK activity [89]. Quercetin exhibited a time- and dose-dependent impact on cell survival in prostate cancer cells [90]. Disturbing the ROS homeostasis and affecting mitochondrial integrity, quercetin caused apoptotic and necrotic cell death in the cells [90]. DU-145 prostate cancer cells with mutated p53 and increased ROS levels displayed a marked decrease in pro-survival AKT pathway activation even though Raf/MEK were stimulated, upon quercetin treatment [90]. However, in the PC-3 prostate cancer cell line, which lacks p53 and PTEN, and bears low ROS levels, vivid activation of AKT and NF-κB pathways were found [90]. Through controlling ROS, AKT, and NF-κB pathways, quercetin employs its anti-cancer impact [90].

Pretreatment with quercetin could make ovarian cancer cells susceptible to radiation-induced cell death in p53 dependent manner [91]; the combinational treatment of irradiation and quercetin could exacerbate DNA damages, cause apoptotic cell death, promote Bax and p21 expressions and reduce Bcl-2 expression in the cells [91]. However, all of these events could be reversed upon knocking down of p53 [91]. The combinational treatment of irradiation and quercetin in the human ovarian cancer xenograft model could restrict tumor growth while activating p53, γ-H2AX and CCAAT/enhancer-binding protein homologous protein [91].

As Caspase-3 is activated in the cancer cells, quercetin ruthenium complex dramatically up-regulates the p53 and Bax, and Caspase-3 expression downregulates the Bcl-2 proteins in HT-29 cell lines [92]. The results of in vivo study showed that the complex treatment could significantly decrease the Bcl-2 expression in colon cancerous tissues while significantly increasing p53 and Bax expression [92]. The results also revealed that the complex caused apoptosis in colon cancer cells via p53-mediated activation of Bax, caspase-3, and Bcl-2 downregulation as well as the suppression of the mTOR/AKT pathway [92]. The vanadium–quercetin combination increased the expression of p53, caspase-9, caspase-3, and Bax and decreased the expression of Bcl-2, mTOR, VEGF, and AKT, resulting in cell cycle arrest and death in rat mammary carcinogenesis and the MCF-7 cell line [93].

When AGS human gastric cancer cells were treated with quercetin, anti-apoptotic Bcl-2, Mcl-1, and Bcl-x protein expression were decreased, and pro-apoptotic proteins such as Bad, Bid, and Bax protein expression was increased [94]. In addition, CDK10 (cyclin-dependent kinase 10), VEGFB (vascular endothelial growth factor B), and KDELC2 (KDEL [Lys-Asp-Glu-Leu] containing 2) gene expressions, associated with apoptosis pathways, were decreased after quercetin treatment [94]. However, quercetin promoted TP53INP1 (tumor protein p53 inducible nuclear protein 1), TNFRSF10D (Tumor necrosis factor receptor superfamily, member 10d, decoy with truncated death domain), and JUNB (jun B proto-oncogene) gene expressions [94, 95].

Quercetin has been shown to promote trichostatin A (TSA) induced apoptosis in H1299 cells, and p53 null cancer cells, in p53-independent manners, which may be less effective than the p53-dependent pathway [79, 96]. The protein expression of p53 significantly was increased in cervical cancer cells including in HeLa, HFF, and SiHa cells after quercetin treatment. Besides, quercetin has also been shown to increase the p53 nuclear signal in SiHa and HeLa cells [97]. Quercetin also increased the transcript level of p21 and the level of Bax protein in HeLa and SiHa cells, famous target genes of p53, suggesting the induction of the transcriptional activity of p53 by quercetin [97]. While increasing p53 and its nuclear signal, quercetin caused apoptosis and cell cycle arrest in the G2 phase in HeLa and SiHa cells [97].

Tamoxifen and quercetin have been reported to control several apoptosis-related gene expressions including p53, Bax, p21, and Bcl-2 in breast cancer cells, leading to cell apoptosis regulation [98]. The results revealed that the impact of quercetin on tamoxifen-induced cell apoptosis is via the p53 signalling pathways [98]. Through suppression of NF-κB and phosphorylation of AKT, the combination of quercetin and curcumin synergistically reduced cell growth and proliferation in K562 chronic myeloid leukemia cells [99]. Additionally, the combination via stimulation of the p53 signalling pathway and FasL triggered apoptotic cell death [99]. Upon exposure to the increasing concentrations of quercetin, p53 expression was increased while cyclin D1 expression was decreased in HepG2 and PANC-1 cells [100]. Furthermore, Quercetin triggered cell cycle arrest in the S phase in GEM-resistant cell lines [100].

Quercetin has been shown to enhance the efficacy of anti-cancer medications such as gemcitabine and doxorubicin, as measured by an increase in the proportion of dead cells [101]. In addition, the results revealed that the combinational treatment of quercetin with anti-cancer drugs decreased the expression of HIF-1α and promoted the expression of cleaved caspase 3 and p53, the regulator of apoptosis [101].

Table 1 presents numerous case studies demonstrating the impact of quercetin on various malignancies via signalling pathways.

**Table 1:** The role of quercetin in various cancers mediated by signalling pathways—evidence from preclinical studies

Signalling pathways	Subfamily involved in the signalling pathway	Cancer types	Quercetin IC_50_	Target genes	Cell line (s)/in vitro model	Possible mechanisms	Refs.
MAPK (family) signalling	p38	Oral cancer	100 µM	MDR1, ABCG2 Hsp27	SCC25	↓ Hsp70 expression changes in EMT ↑apoptosis in drug-resistant cells	[64]
	p38 ERK JNK	Hepatocellular carcinoma	400 µM	–	HepG2 Hep3B	↓growth, ↓proliferation ↑apoptosis cell cycle arrest in the G1 phase	[65]
	p38 JNK ERK	Gastric cancer	267 μM	TRPM7	AGS	↓growth, ↓proliferation, TRPM7 channel inhibition ↑apoptosis	[66]
	p38 ERK1/2 JNK	Choriocarcinoma	20, 50, 100 μM	–	JAR JEG3	↓proliferation cell cycle arrest in the sub-G1 phase ↑ROS, ↑MMP	[67]
	p38 JNK	Gastric cancer	20 and 40 µM	Bcl-2 Bcl-xl Bax	SGC-7901 MGC-803	↓cell viability ↑apoptosis cell cycle arrest in the G2/M phase ↑ROS	[68]
	p38 JNK	Retinoblastoma	0, 25, 75, and 100 µM	p27 p21 Caspase-3 Caspase -9	Y79	↓cell viability cell cycle arrest in the G1 phase ↑apoptosis	[69]
	ERK	Esophageal cancer	0–10 µM	AP-1 NF-κB, p65 COX-2	ESCC	↓growth ↓proliferation ↓inflammation ↓pre-neoplastic lesion formation by NMBA	[70]
	ERK1/2	Prostate cancer	40 μM	p38, ABCG2, NF-κB	PC3, LNCaP ARPE-19	↓ cell viability ↑apoptosis cell cycle arrest in G1 phase ↓cell migration	[71]
	ELK1 MEKK/MAP3K5	Cervical cancer	25, 50, 100 µM	Caspases, pro-apoptotic genes	HeLa	↓growth ↓proliferation ↓colony formation ↑apoptosis ↑cell DNA damage cell cycle arrest in G2/M phase ↓cell migration	[72]
	p38 JNK ERK1/2	Melanoma	0–200 µM	Apoptotic genes	A375SM A375P	↓cell viability ↓growth ↓proliferation ↑morphological and histological changes ↑apoptosis	[73]
	p38 JNK ERK1/2	Canine osteosarcoma	0–100 µM	–	D‐17, DSN	↓proliferation, ↑ MMP, ↑ROS ↓free cytosolic calcium cell cycle arrest in G1 phase	[74]
JAK/STAT (family)	STAT3	Gastric cancer	40 μmol/L	Leptin receptor gene	MGC-803	↑apoptosis ↑necrosis cell cycle arrest in G2/M phase	[53]
	JAK1/STAT3	Glioblastoma	0–100 µM	IL-6 cyclin D1, MMP2	U87, T98G	↓ cancer cells growth ↓ IL-6 ↓Rb phosphorylation, ↓cyclin D1 ↓MMP2 ↓cell migration	[54]
	STAT1/3 JAK1/2	Cholangiocarcinoma	20–100 µM	iNOS, ICAM-1	KKU100, KKU-M139 KKU-M213	↓STAT1/3 phosphorylation ↓iNOS, ↓ICAM-1 ↓growth, ↓migration ↓activity	[55]
	STAT3	Non-small-cell Lung-cancer	10–100 μM	NF-κB, Bcl2 Bax	A549 H460	↓growth ↑apoptosis cell cycle arrest in sub-G1 phase	[56]
	JAK1/STAT3	Breast cancer	0–100 µM	HER-2, MMP-9	BT474	↓ growth and ↓clonogenic ↑apoptosis ↑STAT3	[57]
	JAK2 STAT3/5	Cervical cancer	-	Cyclin D1 Apoptotic proteins	Caski, Hela Siha	↓ cancer cells proliferation, ↓ migration, ↓invasion, ↑apoptosis, ↑autophagy, ↓xenograft growth and development, cell cycle arrest in G2/M phase	[58]
	JAK2/STAT3	Hepatocellular carcinoma	80, 120 μmol/L	–	LM3	↓tumor cell growth ↓viability ↓migration, ↓invasion ↑autophagy cell cycle arrest in S and G2/M phases	[59]
Wnt/β-catenin	β-catenin/Tcf	Teratocarcinoma	70 µM	β-catenin, SOX2, Nanog, Oct4	NT2/D1	↓β-catenin nuclear translocation, ↓transcription factors expression	[37]
	DKK1, 2 and 3	Breast cancer	10, 20, 40 µM	Apoptotic genes	4T1	↑apoptosis ↓ cancer cell viability	[38]
	β-catenin/Tcf	Colon cancer	40, 80 µmol/L	Cyclin D1, survivin	SW480	↑Wnt/β-catenin ↓ cyclin D1, ↓survivin	[39]
	β-catenin/ TCF/LEF	Colon cancer	10–75 µM	GSK3 α ,GSK3 β	HT29	the level of β-catenin in HT29 cells remained unaffected	[40]
PI3K/Akt	p-Akt	Breast cancer	25 µM	PTEN	HCC1937	↓Akt/PKB phosphorylation ↓cell proliferation	[43]
	p-Akt PI3K	Leukaemia	150 µM	Bcl-2, Bax, caspase-2 caspase -3 poly (ADP-ribose) polymerase cleavage	HL-60	cell cycle arrest in G (0)/G (1) phase ↑apoptosis	[44]
	p-Akt	Gastric cancer stem cell	20, 100 µM	Caspase-3 Caspase-9, Bcl2, Cyt-c	MGC803	↑ apoptosis via mitochondrial-dependent pathway and mediated PI3K-Akt signalling pathway	[45]
	PI3K p-Akt	Cervical cancer	25, 50, 100 µM	Bcl-2, Bax	HeLa	cell cycle arrest in G (0)/G (1) phase, anti-proliferative ↑apoptosis	[42]

### The autophagy pathway

Autophagy is the process by which cells, under conditions of starvation and lack of energy, synthesize new macromolecules and ATP through a series of reactions, and maintain normal metabolism and cell survival, respectively [102–104]. Autophagosome formation is an essential step in autophagy and is based on the positive regulator of the ATG1/ULK complex consisting of ATG1, ATG13, and ATG17. The class III PI3K complex is then activated, ATG5-12 conjugated with 16 promoting autophagy membrane elongation, followed by autophagosome LC3II marker formation [105]. Dual membrane autophagosome formation, characterized by PI3 kinase cascade type III-Atg6/Beclin 1, is a major feature of autophagy [106]. Autophagous expansion is performed by two conjugate systems such as ubiquitin: the Atg12-Atg5 conjugate system and the Atg8/LC3-phosphatidyl ethanolamine conjugate system [107]. Signalling autophagy can be combined with multiple signalling pathways in response to various types of cell stress including hypoxia, radiation, starvation, or active chemical insults [108]. From them, the AKT-mTOR classic signalling path is considered as a normal negative regulator to start the formation of two-sided membranes [109], while according to signalling positively, the accumulation of HIF-1α typically activates the progression of autophagy by suppressing the mTOR1 complex or induction of BNIP3/ BNIP3L that can disrupt the interaction of Beclin 1 with Bcl-2/Bcl-xL [110, 111]. The molecular interference between the autophagic signalling pathway and apoptotic is complex and both routes are similar or attached genes that are very important for their respective performance [112]. It has been reported that Atg5, which is essential in the fusion system such as autophagic can be broken in response to death stimuli and then change autophagy to apoptosis [113]. In addition, Bcl-2 and Bcl-xL, two negative apoptosis regulators can connect to the BH3 domain of Beclin 1 and ensure the progress of autophagy [114].

Treatment of stomach cancer cells with quercetin, autophagic vacuoles and acids vaginal organs (AVOs) was developed, LC3I, and A focus on the autophagosome-recruiting LC3II led to the induction of protective autophagy in stomach cancer cells. By decreasing AKT-mTOR signalling and increasing HIF-expression, quercetin prevents the growth of gastric cancer cells [115]. LC3II accumulation and AVOs formation were also found in quercetin-treated glioblastoma cells and colorectal cancer, all of which induced quercetin-protective tumor cell autophagy [116–118]. However, before treatment with chloroquine, an autophagy inhibitor, it can increase apoptosis and inhibit quercetin proliferation [115, 116].

Based on studies, the development of some tumor diseases is strongly related to autophagy, and there are changes in autophagy in a large number of tumor cells. In the oncology field, autophagy initially appears to be a tumor development inhibitor while further research shows that autophagy can upgrade the progression of the tumor [119, 120].

### The Hedgehog signalling pathway

The Discovery of PTCH1 gene mutation in people with Gorlin syndrome and sporadic forms of carcinoma basal cell carcinoma (CBC) has led to the importance of the hedgehog (HH) signalling pathway in human carcinogenesis [121]. Mutations in the PTCH1 gene or one of the components in the hedgehog (HH) signalling pathway is involved in the etiology and the development of some forms of cancers such as—medulloblastoma, breast cancer, colon cancer, pancreatic cancer, esophageal adenocarcinomas and in small cell lung cancer [122]. The hedgehog (HH) signalling pathway is one of the regulatory pathways found in humans preserved since Drosophila Melanogaster. Three counterparts of Drosophila hedgehog (HH) have been identified: Sonic Hedgehog (SHH), Desert Hedgehog (DHH) and Indian Hedgehog (IHH) [123]. Sonic Hedgehog Protein (SHH) is the most important in the development of basal cell carcinoma [124]. PTCH1 is the receptor for all forms of hedgehog (HH) in humans and is part of a cell surface receptor complex consisting of two transmembrane proteins: PTCH1 and SMO (smoothened) [123]. The mechanism by which activation of the hedgehog (HH) pathway leads to carcinogenesis is unknown. Later, hedgehog (HH) is a secretory protein that binds to PTCH1 to activate the signalling pathway. When the hedgehog (HH) binds to the PTCH1 receptor complex, SMO inhibition occurs and the signal is transduced. This is happening through the interaction of a series of proteins, including SUFU (suppressor of fused) which leads to the activation of gli transcription (glioma-associated oncogene) [125]. Gli1 always leads to transcriptional activation, while Gli2 and Gli3 may cause activation or suppression. The regulators of the cell cycle are the target genes WNT, TGF-b, PTCH1 and Gli1. Hedgehog interacting protein (HIP) binds to the hedgehog (HH) and acts as a negative regulator in the signalling pathway [125].

Only a few pharmacological studies are proving the anticancer mechanisms of Quercetin by acting on the hedgehog signalling pathway. In a recent study by Mousavi et al., quercetin nanoparticles were tested in vitro at concentrations of 10, 20, 40, 80, and 100 μM) on LNCaP prostate cancer cells. The results showed that activation of the hedgehog signalling pathway at 40 mM concentrations is the mechanism underlying the anticancer and antiproliferative action [126]. In another study by Slusarz et al., the anticancer effect of Quercetin on PC3 human prostate cancer lines and TRAMP-C2 mouse lines at IC_50_ 1–25 mol/L was demonstrated by inhibiting Gli1 mRNA and hedgehog signalling, respectively [127].

**Fig. 1:**
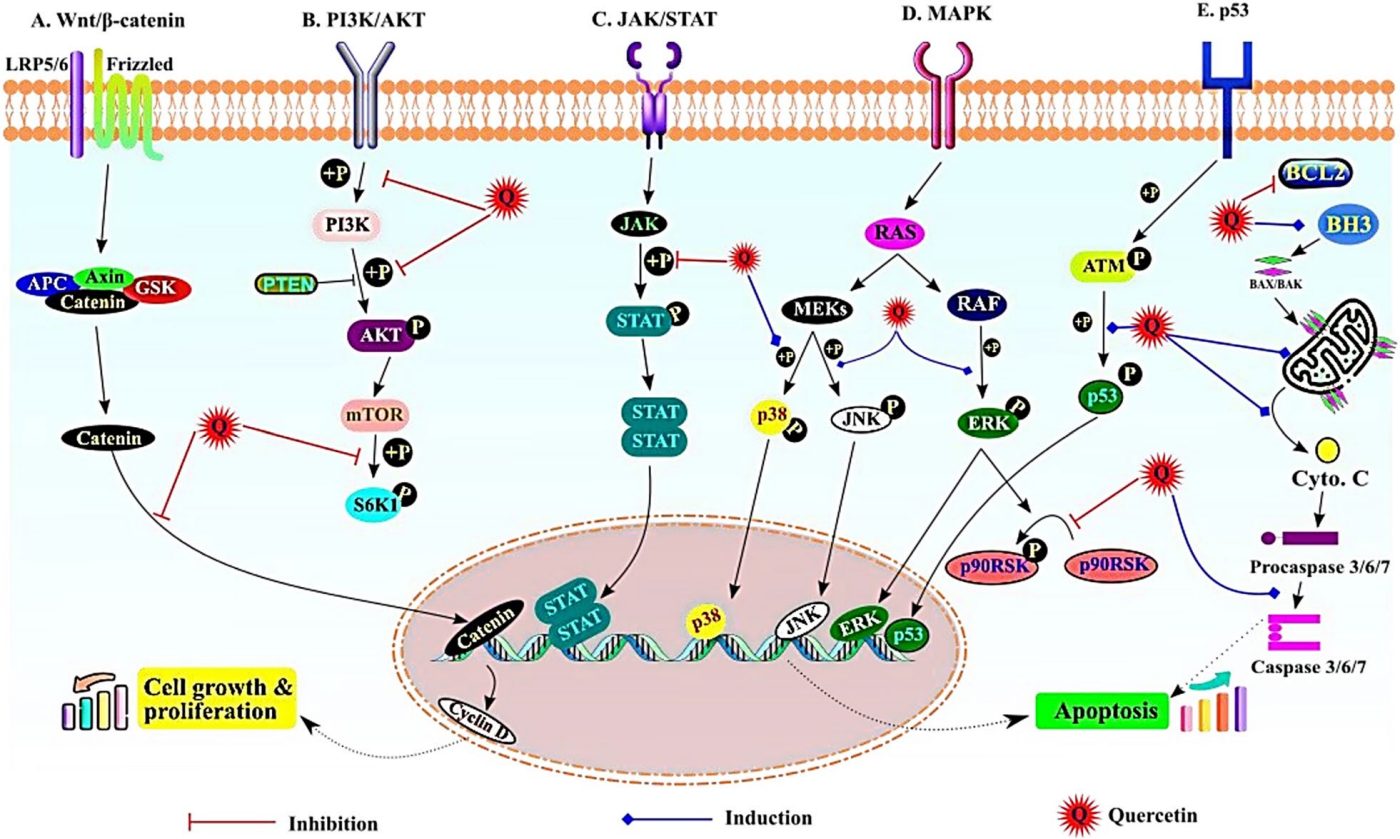
The most important signalling pathways affected by quercetin during cancer prevention. A) Wnt/β catenin pathway; quercetin inhibits the β-catenin translocation in nucleus, B) PI3K/Akt pathway; inhibition of phosphorylation of PI3k, Akt and S6K, C) JAK/STAT pathway; inhibit the p-STAT formation; D) MAPK pathway; induced phosphorylation of p38, JNK and ERK, E) p53 pathway; induced phosphorylation of p53 and induced the apoptosis pathway

The most representative signalling pathways affected by quercetin during cancer prevention are illustrated in Fig. 1.

## Epigenetic modulation by quercetin

### Regulation of miRNAs in different cancer types: effects on cancer cell progression and proliferation

**Fig. 2:**
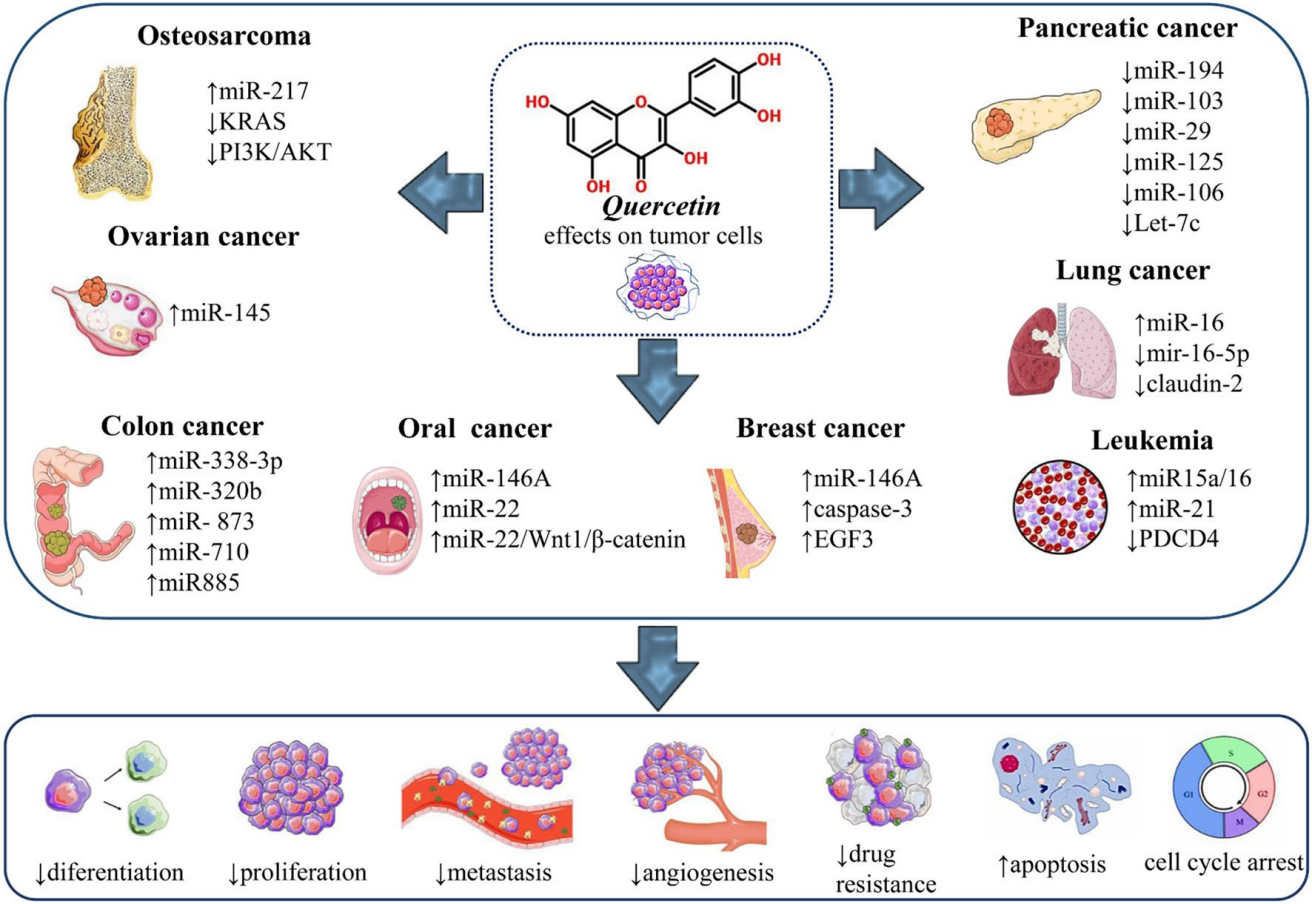
Regulation of miRNAs by quercetin and its role in various cancer types. Abbreviations and symbols: ↑ increase, ↓decrease

Part of the quercetin anticancer effect is exerted by regulating the expression of miRNAs (Additional file 1) and the interaction between quercetin and miRs is an important subject that has been investigated in many studies (Fig. 2).

#### Pancreatic cancer

A recent study on the effect of quercetin on pancreatic cancer shows that the expression of 105 miRNAs changed after treatment with quercetin, including the miR-194, miR-103, miR-29, miR-125, miR-106, and let-7 family, a crucial function in preventing proliferation, invasion, and metastasis as well as promoting cell death is played by these miRNAs. Let-7c is one of the most important miRNAs among them. Numbl is a downstream target of let-7c that is regulated by this miRNA after transcription. The Numbl further antagonizes Notch and therefore prevents the progression of cancer [128]. Numbl and Notch levels are also affected by miR-200b-3p in pancreatic ductal adenocarcinoma (PDA) treatment with quercetin downregulates the miR-200b-3p expression and inhibits self-renewal reduces the cancer stem cells’(CSC) aggressiveness [129]. MiR-142-3p is another example of quercetin-regulated miRs in PDA cell lines [130].

#### Lung cancer

Mir-16 is also influenced by quercetin treatment in lung cancer. Quercetin can elevate the expression level of miR-16. MiR-16 inhibits the expression of claudin-2 in a promoter-independent way by reducing claudin-2 mRNA stability. Claudin-2 is a proliferation driver so this regulation leads to cancer inhibition [131]. Moreover, quercetin treatment increases the radiosensitivity of non-small cell lung cancer (NSCLC) cells by downregulating miR-16-5p and interfering with the miR-16-5p/WEE1 axis [132].

#### Oral cancer

Oral cancer appears as a lesion on the oral mucosa, and is caused by the chaotic division and development of cells; it can develop in any area of the oro-maxillo-facial area, but it most often occurs in the area of the tongue and the floor of the mouth [133]. In oral cancer, quercetin increases miR-16 expression levels, which in turn targets MMP-9, MMP-2 and HOXA10, so quercetin can prevent cell proliferation, migration, and survival in oral cancer [134]. It also has a similar effect on oral squamous cell carcinoma (OSCC). Quercetin has been shown to reduce cell viability and induce apoptosis by positively regulating miR-22 and thus regulating the miR-22/Wnt1/β-catenin axis in OSCC [135].

#### Breast cancer

In a study by Tao at el., it was found that quercetin could positively regulate miR-146a, which in turn, through processes such as regulating the expression of cleaved-caspase-3 and EGFR can induce apoptosis and prevent invasion in breast cancer cells [136].

#### Ovarian cancer

In ovarian cancer cell lines, including SKOV-3 and A2780, quercetin induces apoptosis by rearranging miR-145 [137].

#### Colon cancer

Colon cancer is a type of cancer whose starting point is in the colon, an important segment of the digestive tract [3]. In colon cancer HCT-116 cell line, miR-338-3p -CRC, miR-320b, miR-320c, and miR-320d, miR-125b-2-3p, miR873, miR-710, miR-20, and miR-885 have all been significantly adjusted due to quercetin treatment [138].

#### Osteosarcoma

Quercetin can also regulate the cancer cells' resistance to chemotherapy and radiotherapy. Quercetin-treated osteosarcoma 143B cells show less resistance to cisplatin due to quercetin-induced positive regulation of miR-217. This upregulation decreases one of the miR-217 targets, KRAS level both in mRNA and protein states and inhibits the PI3K/AKT oncogenic pathway and subsequently inhibits the cisplatin resistance [139].

#### Chronic lymphocytic leukemia

Quercetin increases the expression level of miR15a/16 chronic lymphocytic leukemia (CLL) and reduces the radio-resistant B-1 cells’ ability to survive [140]. In addition to the above, quercetin can be effective in counteracting the effects of carcinogens on cells. A 2017 study on BEAS-2B and mouse model showed that quercetin can reverse the upregulation of onco miR-21 and inhibition of its target, tumor suppressor gene programmed cell death 4 (PDCD4), which was induced by hexavalent chromium [Cr(VI)] carcinogen exposure [141] (Fig. 2).

## Quercetin as bioactive molecule in cancer

Plants are important sources for the treatment of cancers because they contain secondary plant metabolites [133, 142, 143]. Quercetin is considered for chemical prophylaxis that acts as a modulator in signal transduction pathways to prevent, inhibit or reverse carcinogenesis [144, 145]. To assess anticancer activities of quercetin in biological systems various studies have been done. Thus, these investigations indicated that quercetin and its metabolites, which exist in diverse plants, have an essential function in protecting against cancer and oxidative stress. As an anti-oxidant and anti-inflammatory, quercetin is widely used. These two quercetin effects have been commonly employed to cope with oxidative stress and inflammation and are shown to be the major source of supplements for individuals suffering from this condition [146].

### Structural activity relationship of quercetin with anticancer mechanism

Quercetin is a flavonoid and chemically it is a phenyl-substituted chromone composed of a basic skeleton of fifteen carbon atoms, composed of a chromium nucleus formed by the benzo ring A and the heterocyclic ring C, and in the aromatic ring B has a phenyl substitution [147]. Data have shown that the different substituents in rings A and B are responsible for the pharmacological activities of quercetin [148].

**Fig. 3:**
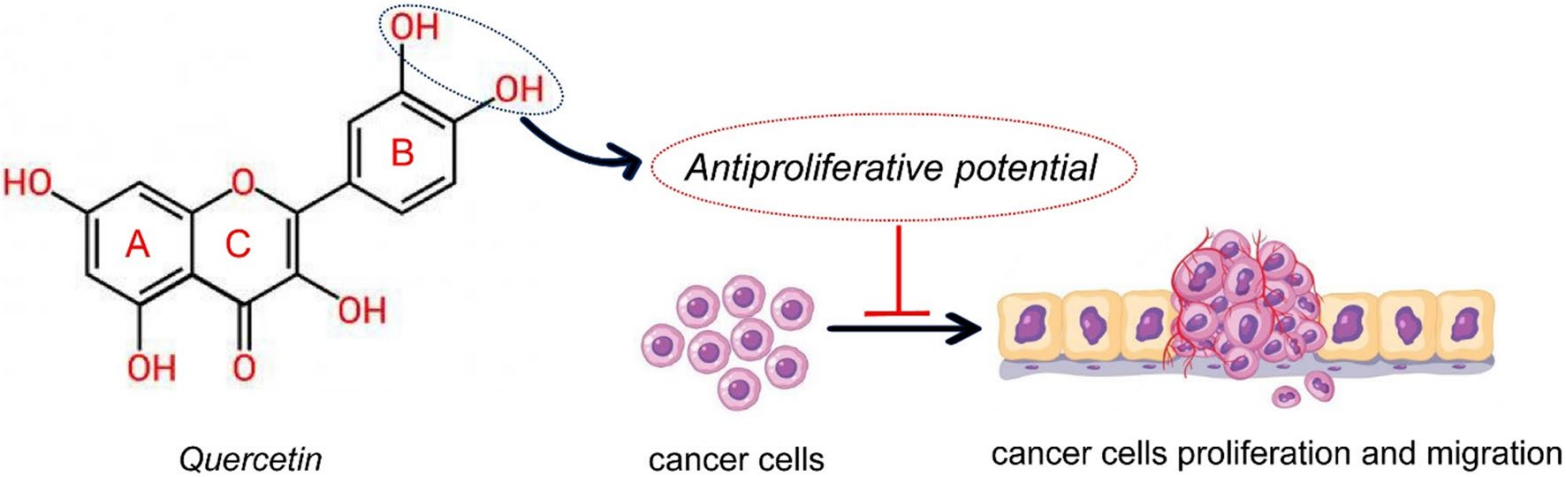
The chemical structure of the flavonol quercetin (3,3′,4′,5,7-pentahydroxyflavone) and potential structure-anticancer activity relationship. Symbol: ⊥ inhibition

Although many researchers have attempted to highlight and understand the possible structure-anticancer activity correlation of quercetin, there is a scarcity of data to explain the relationship between chemical structure-cytotoxic and antitumor effect [149]. As a potential correlation, the hydroxylation pattern of the B ring in the chemical structure of quercetin would be responsible for its antiproliferative effects and inhibition of protein kinase B (AKT) [148, 150] (Fig. 3).

### Synergistic mechanisms of quercetin with anticancer drugs and other phytochemicals

Many studies show that quercetin in combination with anticancer drugs and other anti-cancer bioactive compounds can have a greater effect [151].

The use of quercetin in combination with temozolomide, a standard care chemotherapy treatment for brain tumors, significantly improved the inhibitory effect of temozolomide on human glioblastoma/brain cancer cells and suppressed the survival of glioblastoma cells [152].

The benefits of quercetin in a study by Singhal et al. [153], showed its effects on the management of recurrent cases of breast cancer that is not surgical. In breast cancer cells, quercetin with doxorubicin was administered, and it became clear that the anti-tumor effects of synthetic drugs strengthen. In addition, quercetin combines the two substances to reduce the side effects of synthetic drugs on non-tumor cells. Therefore, it is considered a promising factor in the development of chemotherapy compounds in the treatment of breast cancer [154].

Another study showed that the use of quercetin may increase the anticancer effects of doxorubicin chemotherapy on liver cancer cells while protecting normal liver cells [76]. Li at el. evaluated the impact of the use of quercetin in combination with cisplatin chemotherapy in human oral squamous cell carcinoma (OSCC) cell lines, as well as in oral cancer-induced mice. The study found that the combination of quercetin and cisplatin increased apoptosis in human oral cancer cells, as well as inhibited the growth of cancer in mice, suggesting the therapeutic potential of the combination of quercetin and cisplatin in oral cancer [155].

A combination of hyperoside and quercetin (QH; 1:1) is shown to have synergic anticancer effects. QH treatment in PC3 prostate cancer cell line can downregulate miR-21 expression and therefore, can reduce the inhibitory effect of this miR on its targets PDCD4 and MARCKS, which ultimately leads to decreased invasion and increased apoptosis [156–158]. In addition, QH treatment in renal cancer cells can inhibit the expression of specificity protein (Sp) transcription factors and survive in expression which is controlled by SPs. Further studies show that this inhibition is exerted via down-regulating the oncomiR-27 and up-regulating the zinc finger protein ZBTB10 [157].

Singh et al. demonstrated in vivo in mice with transgenic prostate adenocarcinoma TRAMP that the combination of supplements of quercetin and resveratrol, two antioxidants found abundantly in grapes, had anti-cancer benefits in this mouse model of prostate cancer [159].

The combination of sulforaphane or quercetin with green tea catechins (GTCs) prevents the progression of PDA by upregulating miR-let-7a and downregulating K-ras. The combinations have much more anti-cancer benefits than any of these components alone [160]. Combination of resveratrol and quercetin (RQ) has a similar effect on colon cancer [161].

The synergistic combination of arctigenin and quercetin has been studied in two prostate cancer cell lines, LAPC-4 and LNCaP. The results indicate that this combination can inhibit the expression of oncogenic miRs miR21, miR-19b and miR-148a in LAPC-4 and miR21 and miR-148a in LNCaP cell line way more effectively than quercetin alone [162–164].

### Quercetin and resistance mechanism of anticancer drugs

Multidrug resistance (MDR) is the most important cause of cancer treatment failure. Deciphering the mechanisms of multidrug resistance could contribute to the efficiency of therapeutic strategies for the treatment of neoplastic diseases, and also mitigate the side effects of drugs. [8, 165]. The reason for this is the increased activity of ATP-binding cassette family transporters (ABC) [166].

Quercetin can reverse the resistance mechanism of anticancer drugs through the inhibition of the group P function and ABCB1 gene expression in many cell lines [167, 168]. However, many studies have focused on the anti-cancer properties of this bioactive compound. Several pathways have been identified which can affect quercetin in various cancers [169, 170].

## Therapeutic perspectives

Despite numerous advances in cancer treatment, it is still a life-threatening disease [171–173]. Today, natural compounds due to their predictable function, high therapeutic potential and low toxicity, are significant in preventing various types of cancer [174, 175]. Flavonoids derived from fruits and vegetables are known to be important compounds because of their positive effects on cancer prevention [176].

Various preclinical pharmacological studies have been performed to investigate the anticancer effects of quercetin, which has been shown to reduce the growth rate of cancer cells and also to induce apoptosis [177]. Inducing apoptosis in cancer cells is a vital step to develop a new anticancer drug [146]. In addition, in vivo studies have shown that quercetin is involved in the prevention of several types of cancer, especially colon cancer [178]. Mechanisms responsible for quercetin cancer prevention by eliminating free radicals [179], inhibiting enzymes that activate carcinogens, modifying signal transduction pathways, interacting with estrogen receptors [180], transcription factors [181], and other proteins are affected [182]. Anti-cancer activity of quercetin such as induction of apoptosis [183], fatty acid synthase (FAS) [184], inhibition of cell proliferation [185, 186], reduction of metalloproteinase-2 (MMP-2), and metalloproteinase-9 (MMP-9) expression [187] in prostate cancer cells were studied.

Suppression of carcinogenesis may be due to its radical inhibitory activity [188] and quercetin has been reported to reduce the CYP450 family of enzymes, which plays a key role in the activation of several suspected human carcinogens. Quercetin can demethylase the promoter of the gene p161NK4a, which is hypermethylated in human colon cancer cells [189]. Quercetin regulates the expression of tumor suppressor genes, inhibits the expression of cell cycle genes, and regulates oncogenes expression in the prostate cancer cell line [190]. Quercetin also activates the enzymatic activity of histone deacetylase in which the decreased histone H3 acetylation may be responsible for inhibiting the viable expression and subsequent susceptibility to TRAIL-induced apoptosis.

Studies have revealed that quercetin increases the stability of and promotes the apoptotic effects of the p53 gene through quercetin's ability to phosphorylate and stabilize the p53 gene [191]. In HepG2 cells, quercetin disrupts the cell cycle and induces apoptosis by p53 phosphorylation and by stabilizing p53 in both mRNA and protein levels [192].

Using molecular dynamics simulations, Joshi et al., [193] demonstrated the anti-inflammatory, antioxidant, and analgesic effect observed in the Q-Cl analogue, which indicates good activity in HepG2 cell lines, compared to other cell lines and in particular a decrease in anti-inflammatory activity in structural modification.

Quercetin has been studied in several animal models and human cancer cell lines and found to be present in different cell types including leukemia, colon [194], breast [153], osteosarcoma cells [195], pancreatic [196], gastric [197], and melanoma [198] has anti-proliferative properties. Quercetin has also been tested for leukemia malignancy and has been shown to have an anti-leukemic ability because it acts as an inducer of apoptosis and can also cause allergies [194]. ErbB2 and ErbB3 are tyrosine kinase receptors that have been linked to the growth of human colon cancer and are highly expressed in HT-29 cells. In a study by Kim et al., [194] quercetin reduced the levels of these two enzymes in a dose-dependent manner, and prevented the cell growth of colon cancer cells causing apoptosis in these cells.

Quercetin has been reported to be beneficial against U2OS/MTX300 human osteosarcoma cells by inhibiting proliferation and apoptosis. Inhibition of parathyroid hormone receptor 1 also reduces the invasion, adhesion, proliferation, and migration of osteosarcoma cells [195]. Zhou et al., [196] investigated the effect of quercetin on pancreatic cancer and found that resistance to apoptosis was reversed and that proliferation, angiogenesis, and expression of cancer stem cell markers were reduced by treatment with quercetin, a dietary polyphenol. Quercetin, found freely in foods in addition to forms of β-glycosides such as rutin and quercetin, has been reported to inhibit G1 in human gastric cancer cells [197]. Furthermore, in human gastric cancer cells AGS, quercetin caused morphological changes and reduced total survival through apoptotic cell death, decreased anti-apoptotic proteins Mcl-1, Bcl-2, and Bcl-x and increased the pro-apoptotic proteins Bad, Bax, and Bid [94]. In addition, quercetin has been shown to have anti-cancer effects in melanoma [198]. Inhibition of tumor growth was assessed by quercetin when used as a dietary supplement for experimental models [199].

Clinical therapeutic gaps may result from some potential interactions between quercetin and certain drugs. Oncological patients with various comorbidities who are being treated with antibiotics, anticoagulants, corticosteroids, cyclosporine, digoxin or those on chemotherapy should not take natural supplements with quercetin unless their doctor agrees [200]. Excessive intake of quercetin can lead to side effects, such as digestive effects: nausea, abdominal discomfort or interfering with thyroid function [201].

## Concluding remarks

The plant derivative is an appealing source of alternative anti-tumor drugs. Quercetin, polyphenolic flavonoids, possesses some significant anticancer activities. It has been shown that quercetin uses multiple mechanisms to exert its anticancer effects by modulating different dysregulated signalling pathways which implicated apoptosis and autophagy. Particularly, quercetin exerts its anti-cancer effects via modulating numerous signalling pathways like PI3K/AKT, NF-κB, P53, Wnt/β-catenin, MAPK, JAK/STAT and Hedgehog pathway. Quercetin interferes with numerous intracellular signalling molecules such as TNF-α, Bax, Bcl-2, caspases, and VEGF. The anticancer effects of quercetin have been studied in various types of cancers including breast cancer, prostate cancer, ovarian cancer, lung cancer, colon cancer, hepatocellular carcinoma, lymphoma, and pancreatic cancer. However, the majority of the recent anticancer evidence of quercetin is focused on cancer in vitro models. Due to the lack of in vivo studies, there is an urgent need for different in vivo studies in this field to analyze the therapeutic activities and safety of quercetins. Among natural plant extracts, quercetin shows several significant biological anti-tumor effects. ncRNA (including miRNA and lncRNA) plays a key role in cancer development. Quercetin regulates the expression of ncRNAs, which in turn affects the related signalling pathway genes/proteins expression, suppresses the cancer cell growth, promotes cell apoptosis, and enhances sensitivity to chemotherapy agents. These shed the light on the molecular mechanism between quercetin and ncRNAs which can be considered for its application in clinical adjuvant treatment. Taken together, quercetin is a natural compound with a potential anticancer effect in the adjuvant treatment of cancers.

## Data Availability

Yes.

## Abbreviations

PI3K: Phosphoinositide 3-kinase
AKT: Protein kinase B
JAK: Janus kinase
STAT: Signal transducer and transcription activator
MAPK: Mitogen-activated protein kinase
NF-κB: Nuclear factor kappa B
DKK1: Dickkopf-related protein 1
JAK/STAT: Janus kinase/signal transducers and activators of transcription
ERKs: Stimulated by mitogens and growth factors
GQ: 7-*O*-Granuestine quercetin
MSI: Microsatellite instability
DOX: Doxorubicin
TSA: Trichostatin A
JNK: C-Jun Nterminal kinase
NQ: Nano-quercetin
MET: Methanol extract of triphala
CDK10: Cyclin-dependent kinase 10
VEGFB: Vascular endothelial growth factor B
KDELC2: KDEL [Lys-Asp-Glu-Leu] containing 2
TP53INP1: Tumor protein p53 inducible nuclear protein 1
TNFRSF10D: Tumor necrosis factor receptor superfamily, member 10d, decoy with truncated death domain
JUNB: Jun B proto-oncogene
YYQFT: Yang-Yin-Qing-Fei-Tang
AVOs: Acids vaginal organs
lncRNAs: Long noncoding RNAs
snoRNA: Small Nucleolar RNA
HOTAIR: HOX antisense intergenic RNA
NORAD: Noncoding RNA activated by DNA damage
SAMMSON: The survival associated mitochondrial melanoma specific oncogenic non-coding RNA
PVT1: Plasmacytoma variant translocation 1
MINCR: MYC-induced long non-coding RNA
PCAT1: Prostate cancer associated transcript 1
CCAT1 and CCAT2: Colon and cancer associated transcripts 1 and 2 respectively
TARID: TCF21 antisense RNA inducing demethylation
GAS5: Growth arrest-specific transcript 5
hnRNPK: Heterogeneous nuclear ribonucleoprotein K
BARD1: BRCA1-associated RING domain protein 1
LED: LncRNA activator of enhancer domains
NEAT1: LncRNA nuclear enriched abundant transcript 1
MALAT1: Metastasis associated with lung adenocarcinoma transcript
lncRNA-ATB: LncRNA-activated by TGF-β
SChLAP1: Second chromosome locus associated with prostate-1
PDA: Pancreatic ductal adenocarcinoma
CSC: Cancer stem cells’
NSCLC: Non-small cell lung cancer
OSCC: Oral squamous cell carcinoma
CLL: Chronic lymphocytic leukemia
PDCD4: Programmed cell death 4
QH: Hyperoside and quercetin
Sp: Specificity protein
GTCs: Green tea catechins
RQ: Resveratrol and quercetin
AD: Adenocarcinoma
